# Monitoring Consumption of Common Illicit Drugs in Kuala Lumpur, Malaysia, by Wastewater-Cased Epidemiology

**DOI:** 10.3390/ijerph17030889

**Published:** 2020-01-31

**Authors:** Peng Du, Xin Liu, Guangcai Zhong, Zilei Zhou, Margaret William Thomes, Choon Weng Lee, Chui Wei Bong, Xuan Zhang, Fanghua Hao, Xiqing Li, Gan Zhang, Phong K. Thai

**Affiliations:** 1 Beijing Key Laboratory of Urban Hydrological Cycle and Sponge City Technology, College of Water Sciences, Beijing Normal University, Beijing 100875, China; dup@bnu.edu.cn (P.D.); xuan@bnu.edu.cn (X.Z.); fanghua@bnu.edu.cn (F.H.); 2 Laboratory of Earth Surface Processes, College of Urban and Environmental Sciences, Peking University, Beijing 100871, China; zhouzilei@pku.edu.cn; 3 State Key Laboratory of Organic Geochemistry, Guangzhou Institute of Geochemistry, Chinese Academy of Sciences, Guangzhou 510640, China; liuxin@gig.ac.cn (X.L.); gczhong@gig.ac.cn (G.Z.); 4 Institute of Biological Sciences, University of Malaya, Kuala Lumpur 50603, Malaysia; margaretwt@um.edu.my (M.W.T.); lee@um.edu.my (C.W.L.); cwbong@um.edu.my (C.W.B.); 5 Institute of Ocean and Earth Sciences (IOES), University of Malaya, Kuala Lumpur 50603, Malaysia; 6 Queensland Alliance for Environmental Health Sciences (QAEHS), The University of Queensland, Brisbane 4102, Queensland, Australia; p.thai@uq.edu.au

**Keywords:** substance abuse, MDMA, methamphetamine, ketamine, wastewater analysis, Southeast Asia

## Abstract

Southeast Asian countries including Malaysia play a major role in global drug trade and abuse. Use of amphetamine-type stimulants has increased in the past decade in Malaysia. This study aimed to apply wastewater-based epidemiology for the first time in Kuala Lumpur, Malaysia, to estimate the consumption of common illicit drugs in urban population. Influent wastewater samples were collected from two wastewater treatment plants in Kuala Lumpur in the summer of 2017. Concentrations of twenty-four drug biomarkers were analyzed for estimating drug consumption. Fourteen drug residues were detected with concentrations of up to 1640 ng/L. Among the monitored illicit drugs, 3,4-methylenedioxy-methamphetamine (MDMA) or ecstasy had the highest estimated per capita consumptions. Consumption and dose of amphetamine-type stimulants (methamphetamine and MDMA) were both an order of magnitude higher than those of opioids (heroin and codeine, methadone and tramadol). Amphetamine-type stimulants were the most prevalent drugs, replacing opioids in the drug market. The prevalence trend measured by wastewater-based epidemiology data reflected the shift to amphetamine-type stimulants as reported by the Association of Southeast Asian Nations Narcotics Cooperation Center. Most of the undetected drug residues were new psychoactive substances (NPSs), suggesting a low prevalence of NPSs in the drug market.

## 1. Introduction

According to the United Nations Office on Drug and Crime, both the range of drugs and drug markets are consistently expanding and diversifying more than ever before [1]. Production of opium and manufacturing of cocaine are at the highest levels ever recorded, and markets for cocaine and methamphetamine are extending beyond their usual regions [1]. Southeast Asian countries including Malaysia play a major role in global drug trade and abuse [2,3]. Together with trafficking activities, the use of illicit drugs causes a major problem in Southeast Asian countries, with an increasing rate of drug use in Malaysia [4]. Opioids (i.e., heroin, morphine) continue to be the main drugs of abuse in Malaysia, whereas amphetamine-type stimulants (i.e., ecstasy, methamphetamine) have been recently identified as a growing problem [5]. As a popular club drug, use of ketamine has also increased in recent years [2]. In order to formulate appropriate evidence-based public health and law enforcement policies to protect the public from adverse effects of drug abuse, it is important to have timely and accurate information about the prevalence of drug consumption in the population [6].

Over the last decade, wastewater-based epidemiology, a cost-effective approach to monitor total drug consumption in the population, has been widely applied across Europe [7,8,9,10], North America [11,12], Australia [13,14,15] and Asia [16,17,18]. After years of development, results of wastewater-based epidemiology studies have been adopted as complementary approaches for monitoring drug consumption by authorities in some countries [19]. A synchronized global wastewater-based epidemiology study can provide rapid, objective and up-to-date information to display a world map of drug use [20]. It can be especially useful in supporting drug use evaluation and in comparing different countries and regions from a global perspective. Such vision can be achieved with more wastewater-based epidemiology studies conducted in countries where traditional surveys are difficult to be done. For example, Archer et al. applied the approach to estimate drug use in South Africa as the first wastewater-based epidemiology study on the African continent [21]. Causanilles et al. reported for the first time the estimated drug consumption in Costa Rica, a tropical country of Central America [22]. Subedi et al. carried out the first wastewater-based epidemiology study in India, a South Asian country with the second largest population in the world [23].

Addressing the illicit drug problem is the top priority of Malaysian authorities [24], which requires good estimates of illicit drug consumption as a prerequisite for planning any drug control measures. However, the illicit nature of drug use and the cultural and social stigma against drug addicts in Asia have so far prevented relevant authorities to obtain good estimates of illicit drug consumption in Malaysia. Wastewater-based epidemiology could be used as a complementary monitoring approach, as it provides the total population consumption without revealing any individual information [25]. A recent report on the contemporary drug policy of the region has recommended the use of wastewater-based epidemiology to improve illicit drug demand estimates [26]. However, to the authors’ knowledge, a wastewater-based epidemiology study has not yet been conducted in Malaysia.

Thus, the objective of this work was to obtain, for the first time, a snapshot of the level and profile of illicit drug use in Kuala Lumpur, the capital city of Malaysia, using a wastewater-based epidemiology approach. Wastewater samples were collected from an urban, dense residential community and analyzed for 24 drug biomarkers covering a range of common illicit drugs. Consumption of drugs was estimated and compared with data available in the literature to evaluate the drug use situation in Malaysia.

## 2. Materials and Methods

### 2.1. Reagents and Materials

Standard solutions of twenty-four target analytes and their corresponding deuterated analogs (utilized as internal standards, IS) were purchased from Cerilliant (Round Rock, TX, USA), with details listed in Table S1. The selected targets covered a range of common illicit drugs that are prevalent in Asia, including Malaysia, and most other countries around the world. Having those drugs analyzed in this study facilitated the comparison with previous studies. Formic acid and ammonium formate (HPLC grade) were obtained from CNW Technologies GmbH (Düsseldorf, Germany). HPLC-grade methanol (MeOH) was from Fisher Scientific (Waltham, MA, USA). Hydrochloric acid (AR) and ammonium hydroxide (AR) were purchased from Beijing Chemical Works (Beijing, China). Oasis MCX SPE cartridges (60mg, 3mL) were obtained from Waters Corporation (Milford, MA, USA). Ultrapure water was prepared through a Milli-Q ultrapure system (Millipore, MA, USA).

### 2.2. Sample Collection

Wastewater samples were collected from two wastewater treatment plants (WWTPs) (designated as A and B) in Kuala Lumpur, Malaysia. WWTP-A (101.6706639°E, 3.102744444°N) treats an average of 50,186 m^3^ per day (domestic wastewater, without hospital wastewater) and serves an urban residential community with approximately 220,000 inhabitants. Seven consecutive workdays of influent wastewater samples were collected at WWTP-A in June, July and August, 2017. WWTP-B (101.7403833°E, 3.102788889°N) treats an average of 308 m^3^ per day (hospital wastewater), serving approximately 1400 people. Six consecutive workdays of influent wastewater samples were collected at WWTP-B in July, 2017. WWTP-B serves a hospital where illicit drug consumption is not expected. Therefore, samples from WWTP-B were used as a control for comparing the profile of drug residues with WWTP-A. Samples on weekends were not collected in both WWTPs because entry was denied during this period. Twenty-four-hour time-proportional composite samples were collected through auto-samples (programmed to draw 1 L per hour) in each WWTP. Following collection, the composite samples were acidified to pH 2 by 2M HCl, carried back to the laboratory, and stored at −20 °C until analysis.

### 2.3. Analysis

Sample pretreatment and analysis followed the procedure described in previous publications [27,28] with minor modifications. First, 50 mL wastewater was filtered through a glass fiber membrane to remove solid particles, then spiked with deuterated IS (100 μL, 200 μg/L) before SPE extraction. An Oasis MCX cartridge was conditioned in sequence with 6 mL MeOH, 4 mL ultrapure water (pH = 7) and another 4 mL ultrapure water (pH = 2) at a rate of 1–2 mL/min. The sample was loaded to the conditioned Oasis MCX cartridge under vacuum at the same flow rate. Following loading, the cartridge was washed in sequence with 2 mL ultrapure water (pH = 2) and 2 mL MeOH under vacuum. The cartridge was dried under vacuum, and it was eluted with 4 mL MeOH and 4 mL of 5% NH_3_ in MeOH. The eluate was evaporated until dry by a gentle N_2_ stream, then reconstituted in 400 μL MeOH/ultrapure water (1/5, v/v). The final extract was filtered through a 0.2 μm modified nylon centrifugal filter (VWR International, Radnor, PA, USA) before analysis.

Target analytes were separated using an ultra-fast liquid chromatography (UFLC) system (20 AD-XR, Shimadzu, Japan) with a Phenomenex Gemini C18 column (100 × 2 mm, 3 μm). The mobile phase was composed of 30 mM ammonium formate in ultrapure water, with pH adjusted to 3.5 by formic acid (A) and MeOH (B). The elution gradient was as follows: 0–0.1 min, 5% B; 0.1–3.0 min, 30% B; 3.0–5.0 min, 80% B; 5.0–5.5 min, 95% B; 5.5–9.5 min, 95% B; 9.5–9.6 min, 5% B; 9.6–14.0 min, 5% B (Figure S1). The injection volume was 5 μL, and the flow rate of the mobile phase was controlled at 0.3 mL/min. Concentrations were determined using an API-4000 triple quadrupole mass spectrometer (AB SCIEX, Boston, MA, USA) equipped with an electrospray interface operating in positive ionization mode. The quantification of the mass spectrometry (MS) system was operated in multiple reaction monitoring (MRM) mode. Details of MS parameters (declustering potential, collision energy, quantifier and qualifier ions), IS and retention time are described in Table S2.

The analytical procedures were subjected to strict quality control and quality assurance measures. The limit of detection (LOD), limit of quantification (LOQ), recoveries, matrix effects, repeatability and reproducibility were examined according to previously established protocol [29]. The recoveries and matrix effects of target compounds ranged from 83.6% ± 10.1% to 104.9% ± 6.2% and from −10.1% ± 6.6% to 17.2% ± 7.3%, respectively. Procedure blanks using ultrapure water (pH = 2) spiked with IS were included in every 10th sample for checking the potential interference and contamination, and all target analytes were below LOD in blanks. More details can be found in the Supplementary Materials (Table S3).

### 2.4. Mass Load and Consumption Calculation

The daily mass load of each drug residue per 1000 inhabitants at a specific WWTP was estimated by Equation (1). *C_i_* is the influent concentration of the target drug residue, *F_In_* is the influent flow rate of the specific WWTP and *PS* is the population served by the WWTP.

(1) $$Load\;(mg/1000\;inh/d)=\frac{C_{i}{(ng/L)\times F}_{In}\left(L/d\right)}{\frac{PS}{1000}}\times \frac{1}{10^{6}}(\frac{mg}{ng})$$

(2) $$Consumption\;=Load\times \frac{{MW}_{pi}}{{MW}_{mi}}\times \frac{1}{{EF}_{i}}$$

The consumption (mg/1000 inh/d) of target drug was estimated by Equation (2), where *EF_i_* is excretion factor of a given dose of target drug excreted as unchanged parent or metabolite through urine, *MW_pi_* is the molecular weight of the parent, and *MW_mi_* is the molecular weight of the metabolite. The human excretion factors of the target drug are shown in Table S4. Uncertainties involved in the above estimation process have been discussed in previous studies [30,31].

### 2.5. Statistical Analysis

Statistical analysis was performed via SPSS 20 (IBM Co., Armonk, NY, USA), and the difference was statistically significant with a *p*-value below 0.05. The Kolmogorov–Smirnov (K-S) test was employed for a normal test before other analyses. Pearson correlation analysis was used to assess the correlation between loads of the unchanged parent and metabolite. Student’s *t*-test was used to compare the differences of mass loads between WWTP-A and -B.

## 3. Results and Discussion

### 3.1. Occurrence and Daily Mass Loads of Drug Residues in Influents

Fourteen among 24 target drug biomarkers were detected in the influent samples of WWTP-A, with the concentrations ranging from <LOD (6-acetylmorphine and 3,4-methylene-dioxyamphetamine (MDA)) to 1640 ng/L (methamphetamine) (Table 1). Methamphetamine had the highest mean concentration (1014 ± 246 ng/L), followed by 3,4-methylenedioxymethamphetamine (MDMA) (812 ± 346 ng/L), ketamine (274 ± 39 ng/L) and tramadol (185 ± 30 ng/L). The mean concentrations of other drug residues were all below 100 ng/L. In the small WWTP-B, only eleven drug residues were detected, with the mean concentrations ranging from 6 ± 9 ng/L (morphine) to 639 ± 95 ng/L (tramadol) (Table 1).

A strong, positive correlation (*p* < 0.001) was found between influent amphetamine and methamphetamine concentrations in WWTP-A, and the mean ratio was 0.068 ± 0.016. These low ratios (<0.1) indicated that amphetamine detected in the samples mainly came from methamphetamine use, not from amphetamine use itself [32,33]. Positive correlations (*p* < 0.05) were also found between cocaine and benzoylecgonine, 2-ethylidene-1,5-dimethyl-3,3-diphenylpyrrolidine (EDDP) and methadone, and ketamine and norketamine; the mean ratios were 0.35 ± 0.19, 3.60 ± 1.12 and 3.03 ± 0.43, which was consistent with previous reports in wastewater [34]. This suggested that the drug residues measured in our samples were primarily from human consumption rather than from random dumping. Emerging illicit drugs such as cathinone, p-methoxymethamphetamine, methylone, mephedrone, 4-iodo-2,5-dimethoxyphenethylamine, 3,4-methylenedioxypyrovalerone, benzylpiperazine, 3-trifluoromethylphenylpiperazine, 1-(3-chloro-phenyl) piperazine and low-dose fentanyl were not detected. The results indicated that the use of these new psychoactive substances was not as prevalent as the other common illicit drugs in the studied area.

In WWTP-A, methamphetamine, MDMA, ketamine and tramadol had higher mean mass loads than other drugs, with the values of 231 ± 56 mg/1000 inh/d, 185 ± 79 mg/1000 inh/d, 62 ± 9 mg/1000 inh/d and 42 ± 7 mg/1000 inh/d, respectively (Table 2). For WWTP-B, tramadol had the highest influent mean load (144 ± 21), followed by ketamine (62 ± 5 mg/1000 inh/d) and methamphetamine (30 ± 16 mg/1000 inh/d) (Table 2). The profile of drug residues in WWTP-A was different from that in WWTP-B, which was attributed to different sources of influents from WWTP-A (domestic wastewater) and WWTP-B (wastewater from a hospital). It was reasonable that the loads of the pain-killer tramadol were much higher in samples from WWTP-B, while the loads of illicit drugs were higher in WWTP-A (*p* < 0.05).

### 3.2. Estimation of Community Drug Consumption

For the discussion of illicit drug consumption in the community, only data from WWTP-A were used, as they are representative of a large population of Kuala Lumpur where people could have easier access to drug and locations for drug use than inside the hospital served by WWTP-B.

Our monitoring study estimated the consumption of common illicit drugs such as MDMA, methamphetamine, ketamine, cocaine, heroin as well as prescribed drugs prone to abuse such as codeine, tramadol and methadone. As shown in Table 3, MDMA, methamphetamine and ketamine were the three most popular illicit drugs consumed in this population. This finding was in agreement with the recent report in which methamphetamine, MDMA and ketamine were listed as synthetic drugs of concern in the region [26].

**Figure 1:**
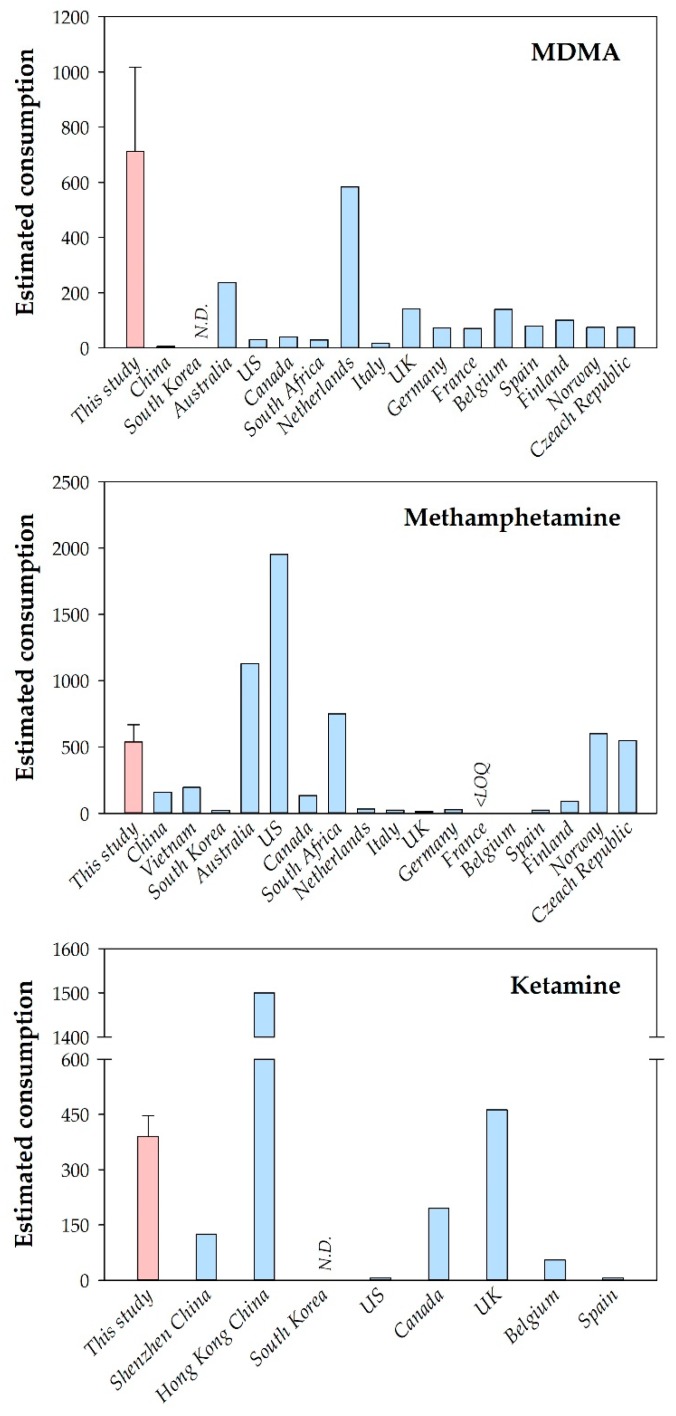
Estimated methamphetamine, MDMA and ketamine consumptions (mg/1000 inh/d) in this study and other countries [3,12,35,36,37,38,39,40,41,42,43,44,45,46].

In this study, MDMA had the highest estimated per capita consumption, ranging from 558 ± 373 mg/1000 inh/d (July) to 850 ± 177 mg/1000 inh/d (August) in WWTP-A (Table 3). The mean MDMA consumption in this study was much higher than those reported in other countries (Figure 1). It was noteworthy that the MDMA consumption estimated in Kuala Lumpur, Malaysia, was even higher than the Netherlands, the country with the highest MDMA consumption in the latest Sewage Analysis CORe group Europe study, which reported the temporal and spatial consumption trend of common illicit drugs, including MDMA, in >60 cities around the world [35]. This result suggested that Malaysia is not only a substantial point of entry for MDMA to the regional market [26] but also a large consumer of this drug.

The drug with second highest consumption in Kuala Lumpur was methamphetamine with mean consumption ranging from 468 ± 64 mg/1000 inh/d (July) to 687 ± 112 mg/1000 inh/d (August) (Table 3). Consumption of ketamine, a popular drug of abuse in Southeast Asia [21], was also relatively high after methamphetamine, ranging from 357 ± 64 mg/1000 inh/d (June) to 434 ± 40 mg/1000 inh/d (August) (Table 3). Consumption of methamphetamine and ketamine measured by wastewater-based epidemiology in Kuala Lumpur was also higher than in most cities around the world (Figure 1). These results indicated that synthetic drugs were highly prevalent in the city. It could be explained by the fact that Kuala Lumpur is located in one of the most important trafficking routes of synthetic drugs in Southeast Asia [21,22]. In general, the profile of illicit drug consumption of Kuala Lumpur was different to that of other cities around the world. For example, although the levels of methamphetamine consumptions were similar between this study and two cities in South Africa [18], the prevalence of MDMA was significantly higher in Kuala Lumpur, while cocaine consumption was popular in the South African cities (Figure 1).

Heroin and codeine are two traditional opiates. Consumption of codeine ranged from 22 ± 6 mg/1000 inh/d (July) to 26 ± 8 mg/1000 inh/d (June). It can be metabolized into morphine by the human body. In this study, the influent codeine loads were around 7 mg/1000 inh/d, and the mean load of morphine from codeine metabolized was about 1 mg/1000 inh/d (based on the excretion rates of 50% and 9%) [47]. Thus, we assumed the morphine measured in the influent samples was from heroin consumption because the morphine from actual codeine consumption was within the range of measurement error of morphine loads. Meanwhile, morphine was not mentioned as a substance of abuse in any reports about illicit drugs in Malaysia. This assumption will get an overestimated value, but it is acceptable within the allowable range of error. Hence, the estimated heroin consumption was in the range from 38 ± 8 mg/1000 inh/d to 51 ± 11 mg/1000 inh/d, respectively. Consumption of methadone and tramadol ranged from 14 ± 7 mg/1000 inh/d to 152 ± 21 mg/1000 inh/d (Table 3).

For cocaine, the wastewater-based epidemiology-estimated consumption ranged from 9 ± 4 mg/1000 inh/d to 14 ± 6 mg/1000 inh/d. Obviously, it was not as prevalent as the mentioned drugs in Kuala Lumpur, and the low consumption was consistent with the low levels of seizures cocaine in Malaysia [48].

In 2017, traditional surveys showed that the prevalence of drug abuse in Malaysia has been shifting from opiates to amphetamine-type stimulants [24]. The number of users of methamphetamine and MDMA have substantially increased while that of opiate users has decreased, especially heroin users (Table S5) [24]. Furthermore, recent data of amphetamine-type stimulants showed seizures were two orders of magnitude higher than that of opiates and synthetic opioids in recent years [23,48]. However, the survey and seizure data showed indirect and delayed information of drug use rather than the actual drug consumption [49]. Wastewater-based epidemiology estimated that amphetamine-type stimulant (methamphetamine and MDMA) consumption and doses were both an order of magnitude higher than those of opioids (heroin and codeine, methadone and tramadol), even if the consumption of heroin was overestimated (Table 3). The wastewater-based epidemiology-estimated profile of drug use reflected the changing profile of drug use and trafficking recorded by the traditional methodologies in Malaysia. This result implied that wastewater-based epidemiology could assess the prevalence and consumption of drug use more specifically, objectively and in real-time, which in turn indicated a good response of wastewater-based epidemiology to the change of profile of drug use compared to traditional monitoring approaches.

### 3.3. Limitations

The limitations of the back-estimation process by wastewater-based epidemiology have been discussed in detail elsewhere [30,50,51]. Most notably, wastewater-based epidemiology cannot provide information on prevalence and frequency of use, characteristics and types of consumers as well as the purity of drugs. Illegal synthesis processes used for the manufacturing of these drugs or dumping can also overestimate the final estimates if the parent compounds are used as biomarkers for back-estimation.

Because of the limited numbers of samples and WWTPs, the findings in this study can be considered as preliminary for the urban area of Kuala Lumpur. Further research in the field should be conducted to get spatial-temporal variations and involve more communities in Southeast Asian countries for longer sampling periods.

## 4. Conclusions

Using wastewater-based epidemiology, this study provides the first objective snapshot of local drug use in a population of Kuala Lumpur, Malaysia. Fourteen drug residues were detected with concentrations of up to 1640 ng/L in influents. MDMA had the highest estimated per capita consumption, and it was higher than most other countries around the world. The amphetamine-type stimulants (methamphetamine and MDMA) were the most prevalent drugs, replacing opioids in the drug market. The prevalence trend measured by wastewater-based epidemiology data reflected the shift to amphetamine-type stimulants, as reported by the traditional survey data in Malaysia. This study can guide and promote future wastewater-based epidemiology monitoring in Southeast Asia, and it can provide additional understanding of the drug market for the authorities.

## Figures and Tables

**Table 1:** Statistics of drug residue concentrations (ng/L) in wastewater treatment plant (WWTP)-A and -B.

Drug Residues	WWTP-A (*n* = 20)					WWTP-B (*n* = 6)			
	DF ^a^ (%)	Range	Mean ± STD ^b^	Median		DF (%)	Range	Mean ± STD	Median
Methamphetamine	100	690–1640	1014 ± 246	956		100	47–225	132 ± 72	118
Amphetamine	100	47–93	69 ± 15	73		100	<LOQ–22	8 ± 7	5
Ketamine	100	188–354	274 ± 39	284		100	243–311	276 ± 24	279
Norketamine	100	51–106	91 ± 14	94		100	146–206	175 ± 20	174
Morphine	100	35–93	65 ± 14	67		33	<LOD–22	6 ± 9	<LOD
Codeine	100	18–45	32 ± 8	31		33	<LOD–30	9 ± 14	<LOD
6-acetylmorphine	20	<LOD–13	2 ± 4	<LOD		0	<LOD	<LOD	<LOD
Cocaine	100	1–11	6 ± 3	5		0	<LOD	<LOD	<LOD
Benzoylecgonine	100	6–35	19 ± 8	16		0	<LOD	<LOD	<LOD
MDMA	100	290–1296	812 ± 346	936		100	5–52	54 ± 59	28
MDA	95	<LOD–51	28 ± 10	29		100	<LOQ–91	53 ± 29	60
Methadone	100	<LOQ–31	10 ± 7	10		100	<LOQ–13	8 ± 4	8
EDDP	100	3–56	31 ± 17	37		100	19–25	23 ± 3	23
Tramadol	100	146–265	185 ± 30	185		100	490–757	639 ± 95	634

^a^ DF—detection frequency; ^b^ STD—standard deviation.

**Table 2:** Mean influent loads (mg/1000 inh/d) of drug residues in WWTP-A and -B.

Drug Residues	WWTP-A			WWTP-B
	June	July	August	July
Methamphetamine	207 ± 38	201 ± 27	295 ± 48	30 ± 16
Amphetamine	15 ± 3	14 ± 3	19 ± 2	2 ± 2
Ketamine	57 ± 10	62 ± 6	69 ± 6	62 ± 5
Norketamine	19 ± 3	21 ± 3	23 ± 1	39 ± 5
Morphine	17 ± 4	12 ± 3	16 ± 2	1 ± 2
Codeine	8 ± 2	7 ± 2	7 ± 1	2 ± 3
6-acetylmorphine	<1	<1	1 ± 1	<1
Cocaine	1 ± 1	1 ± 1	2 ± 1	<1
Benzoylecgonine	4 ± 12	3 ± 2	5 ± 2	<1
MDMA	195 ± 73	145 ± 97	221 ± 46	12 ± 13
MDA	6 ± 3	7 ± 2	6 ± 1	12 ± 7
Methadone	2 ± 1	2 ± 2	2 ± 1	2 ± 1
EDDP	7 ± 3	7 ± 4	7 ± 5	5 ± 1
Tramadol	44 ± 9	40 ± 5	44 ± 6	144 ± 21

**Table 3:** Estimated community consumption (mg/1000 inh/d) and mean dose (dose/1000 inh/d) of drugs serviced by WWTP-A.

Drugs	June	July	August
MDMA	748 ± 282 ^a^ (7.5) ^b^	558 ± 373 (5.6)	850 ± 177 (8.5)
Methamphetamine	481 ± 88 (16.0)	468 ± 64 (15.6)	687 ± 112 (22.9)
Ketamine	357 ± 64 (4.8)	387 ± 35 (5.2)	434 ± 40 (5.8)
Cocaine	14 ± 6 (0.1)	9 ± 4 (0.1)	12 ± 4 (0.1)
Tramadol	150 ± 32 (3.0)	137 ± 17 (2.7)	152 ± 21 (5.1)
Methadone	14 ± 7 (0.6)	15 ± 9 (0.6)	15 ± 9 (0.6)
Codeine	26 ± 8 (0.7)	22 ± 6 (0.6)	24 ± 5 (0.6)
Heroin ^c^	51 ± 11 (3.4)	38 ± 8 (2.5)	48 ± 5 (3.2)

^a^ Consumption (Mean ± STD); ^b^ Mean dose; ^c^ Assumed the morphine in influents was all coming from heroin abuse.

## Supplementary Materials

The following are available online at https://www.mdpi.com/1660-4601/17/3/889/s1, Table S1: Target analytes and corresponding internal standers, Table S2: MS parameters (quantifier and qualifier ions), declustering potential, collision energy and retention time, Table S3: Method validation parameters: recovery, matrix effect, reproducibility, reproducibility, LOD, LOQ and procedure bank, Table S4: The human excretion factors of the target drugs, molecular weight ratio of parent and metabolite and typical dose, Table S5: Number of drug dependents of amphetamine-type stimulants and opiates in Malaysia from 2013–2017, Figure S1: The elution gradient of mobile phase B (MeOH), Figure S2: Chromatogram of 24 analyzed substances and their corresponding deuterated.
