# Supplementary material for: Monitoring Consumption of Common Illicit Drugs in Kuala Lumpur, Malaysia, by Wastewater-Cased Epidemiology
Source: Int J Environ Res Public Health. 2020 Jan 31;17(3):889. doi: 10.3390/ijerph17030889 (PMC7036889; doi:10.3390/ijerph17030889)
Supplement: Supplementary file 1 [file ijerph-17-00889-s001.pdf]

Table S1. Target analytes and corresponding internal standers.

Analyte	Internal Standard
Methamphetamine	Methamphetamine-d8
Amphetamine	Amphetamine-d8
3,4-Methylenedioxymethamphetamine	3,4-Methylenedioxymethamphetamine-d5
3,4-Methylene-dioxyamphetamine	3,4-Methylene-dioxyamphetamine -d5
Ketamine	Ketamine-d4
Norketamine	Norketamine-d4
6-Acetylmorphine	6-Acetylmorphine-d6
Morphine	Morphine-d3
Codeine	Codeine-d6
Methadone	Methadone-d9
2-Ethylidene-1,5-dimethyl-3,3-diphenylpyrrolidine	2-Ethylidene-1,5-dimethyl-3,3-diphenylpyrrolidine-d3
Cocaine	Cocaine-d3
Benzoylecgonine	Benzoylecgonine-d3
Cathinone	Cathinone-d5
Mephedrone	Mephedrone-d3
p-Methoxymethamphetamine	p-Methoxymethamphetamine-d3
Benzylpiperazine	Benzylpiperazine-d7
1-(3-chlorophenyl) Piperazine	1-(3-chlorophenyl) Piperazine-d8
3-Trifluoromethylphenylpiperazine	3-Trifluoromethylphenylpiperazine-d4
4-Iodo-2,5-Dimethoxyphenethylamine	4-Iodo-2,5-Dimethoxyphenethylamine-d3
Tramadol	Tramadol-^{13}C-d3
Fentanyl	Fentanyl-d5
Methylone	Methylone-d3
3,4-Methylenedioxypyrovalerone	3,4-Methylenedioxypyrovalerone-d8

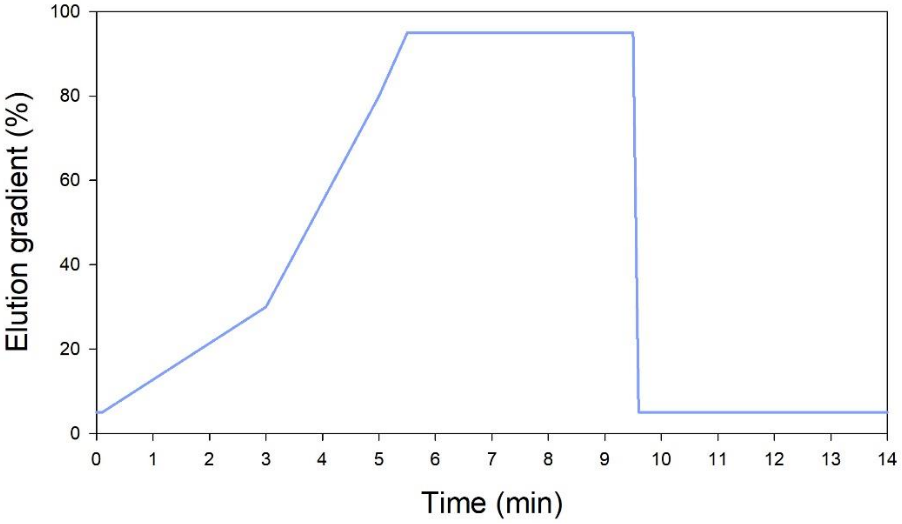

Figure S1. The elution gradient of mobile phase B (MeOH).

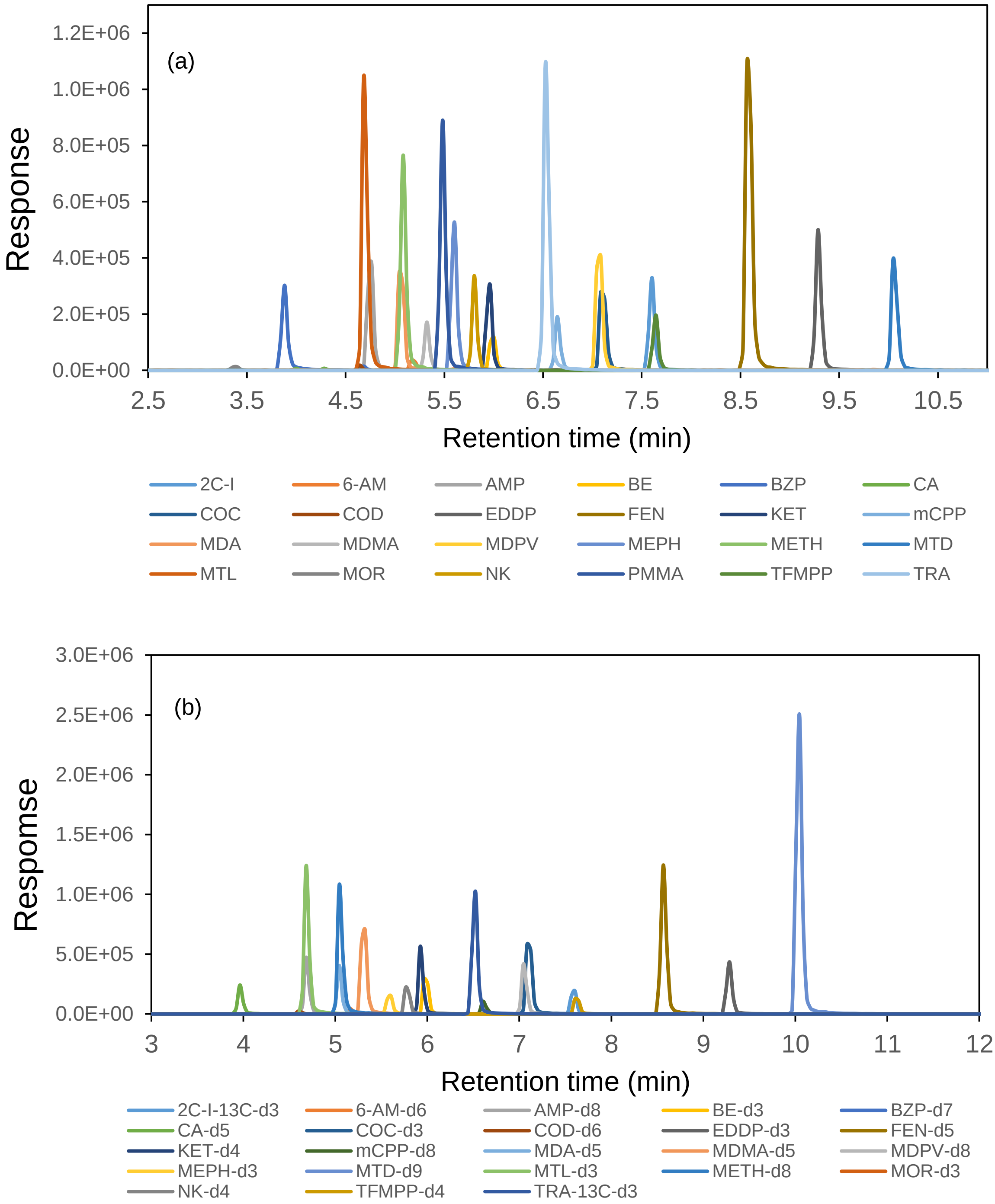

Figure S2. Chromatogram of 24 analyzed substances (**a**) and their corresponding deuterated (**b**).

Table S2. MS parameters (quantifier and qualifier ions), declustering potential, collision energy and retention time.

Analyte	Retention time (min)	Precursor Ion (m/z)	Declustering Potential (V)	Quantifier		Qualifier	
				Product Ion (m/z)	Collision Energy (V)	Product Ion (m/z)	Collision Energy (V)
Methamphetamine	5.08	150.1	35	119.2	15	91.2	20
Amphetamine	4.76	136.1	40	119.1	15	91.2	20
Ketamine	5.96	238.1	50	207.1	20	125.1	38
Norketamine	5.80	224.1	40	125.1	32	207.1	16
Morphine	3.40	286.2	90	165.1	50	201.3	35
Codeine	4.64	300.1	108	199.1	40	165.1	54
6-Acetylmorphine	5.16	328.1	90	165.2	49	211.1	35
Cocaine	7.08	304.1	114	182.2	26	150.3	34
Benzoylecgonine	6.00	290.1	107	168.1	26	105.1	40
3,4-Methylenedioxymethamphetamine	5.32	193.8	70	162.9	15	134.9	25
3,4-Methylene-dioxyamphetamine	5.04	180.1	41	162.9	15	135.0	26
Methadone	10.05	310.3	110	265.1	20	105.1	40
2-Ethylidene-1,5-dimethyl-3,3-diphenylpyrrolidine	9.29	278.2	100	234.2	33	249.2	33
p-Methoxymethamphetamine	5.48	180.2	50	149.2	23	121.2	30
Methylone	4.68	208.2	55	160.0	25	190.1	16
4-Iodo-2,5-Dimethoxyphenethylamine	7.60	308.2	55	291.0	20	275.8	30
Mephedrone	5.60	178.1	80	160.0	19	145.2	29
Cathinone	4.28	150.0	80	132.1	19	117.1	32
3,4-Methylenedioxypyrovalerone	7.08	276.1	80	126.1	34	135.0	38
Benzylpiperazine	3.88	177.1	80	91.0	32	85.1	22
3-Trifluoromethylphenylpiperazine	7.64	231.0	80	188.0	33	119.0	42
1-(3-chlorophenyl) Piperazine	6.64	197.0	80	154.1	27	119.2	35
Tramadol	6.52	264.4	50	58.1	30	246.3	15
Fentanyl	8.57	337.4	50	188.1	30	216.1	30
Methamphetamine-d8	5.04	158.1	40	124.2	16	-	-

Amphetamine-d8	4.68	144.1	40	127.1	13	-	-
3,4-Methylenedioxymethamphetamine-d5	5.32	199.1	65	165.0	17	-	-
3,4-Methylene-dioxyamphetamine-d5	5.04	185.1	55	168.0	15	-	-
Ketamine-d4	5.92	242.1	60	129.1	37	-	-
Norketamine-d4	5.76	228.1	45	129.1	30	-	-
6-Acetylmorphine-d6	5.16	334.2	90	211.2	36	-	-
Morphine-d3	3.44	289.1	90	181.1	51	-	-
Codeine-d6	4.60	306.2	103	165.2	55	-	-
Methadone-d9	10.05	319.3	60	268.2	22	-	-
2-Ethylidene-1,5-dimethyl-3,3-diphenylpyrrolidine-d3	9.29	281.2	100	234.2	33	-	-
Cocaine-d3	7.08	307.1	105	185.1	28	-	-
Benzoylecgonine-d3	5.96	293.2	93	171.2	27	-	-
Cathinone-d5	3.96	155.3	50	137.1	16	-	-
Mephedrone-d3	5.60	181.1	80	163.0	19	-	-
Benzylpiperazine-d7	7.08	184.1	80	98.0	32	-	-
1-(3-chlorophenyl) Piperazine-d8	6.60	205.1	80	158.1	31	-	-
3-Trifluoromethylphenylpiperazine-d4	7.60	235.1	80	190.0	32	-	-
4-Iodo-2,5-Dimethoxyphenethylamine-^{13}C-d3	7.60	312.3	53	294.9	19	-	-
Fentanyl-d5	8.57	342.3	90	187.9	30	-	-
Tramadol-^{13}C-d3	6.52	268.4	50	58.1	40	-	-
Methylone-d3	4.68	211.3	50	162.9	23	-	-
3,4-Methylenedioxypyrovalerone-d8	7.04	284.1	80	134.2	39	-	-

Table S3. Method validation parameters: recovery, matrix effect, repeatability, reproducibility, LOD, LOQ and procedure bank.

Analyte	Recovery	Matrix	Repeatability	Reproducibility	LOD [a]	LOQ [b]	Procedure Bank
	400 ng/L (*n* = 3) (%)		10 μg/L (*n* = 5) (RSD %) [c]		ng/L	ng/L	ng/L (*n* = 5)
Methamphetamine	92.2 ± 4.2	−5.5 ± 8.8	2.3	4.3	0.2	0.8	<LOD
Amphetamine	98.7 ± 5.5	−2.1 ± 0.9	5.8	8.8	2.0	4.0	<LOD
Ketamine	97.6 ± 5.5	−3.0 ± 2.3	4.3	5.8	0.2	0.8	<LOD
Norketamine	98.2 ± 6.2	4.9 ± 5.8	2.5	3.9	2.0	0.8	<LOD
Morphine	95.6 ± 13.6	−6.1 ± 4.7	1.6	6.1	0.5	2.0	<LOD
Codeine	95.3 ± 6.2	0.2 ± 10.0	4.9	9.1	0.5	2.0	<LOD
6-Acetylmorphine	83.6 ± 10.1	17.2 ± 7.3	3.6	3.6	0.5	0.8	<LOD
Cocaine	95.1 ± 8.2	−4.2 ± 2.0	4.8	5.5	0.2	0.8	<LOD
Benzoylecgonine	96.1 ± 3.6	−6.3 ± 1.3	2.0	3.7	0.2	0.8	<LOD
3,4-Methylenedioxymethamphetamine	104.9 ± 6.2	10.9 ± 8.3	3.4	3.3	0.2	0.8	<LOD
3,4-Methylene-dioxyamphetamine	90.2 ± 5.5	−5.8 ± 2.1	0.5	1.4	2.0	4.0	<LOD
Methadone	102.4 ± 4.1	−1.2 ± 5.5	1.2	5.1	0.2	0.8	<LOD
2-Ethylidene-1,5-dimethyl-3,3-diphenylpyrrolidine	104.9 ± 3.8	0.6 ± 4.1	3.9	4.0	0.2	0.8	<LOD
p-Methoxymethamphetamine	86.4 ± 4.8	−10.3 ± 3.5	2.8	6.4	2.0	4.0	<LOD
Methylone	104.8 ± 7.6	2.2 ± 4.0	3.9	2.5	0.2	0.8	<LOD
4-Iodo-2,5-Dimethoxyphenethylamine	100.2 ± 7.0	−3.0 ± 1.7	6.9	8.7	0.2	0.8	<LOD

Mephedrone	101.2 ± 3.7	5.3 ± 3.5	1.1	5.9	0.2	0.8	<LOD
Cathinone	99.6 ± 11.7	−7.2 ± 2.5	2.3	2.4	0.2	0.8	<LOD
3,4-Methylenedioxypyrovalerone	102.7 ± 5.1	6.2 ± 2.6	6.1	7.1	0.2	0.8	<LOD
Benzylpiperazine	88.2 ± 9.7	8.3 ± 9.6	6.3	14.8	0.2	0.8	<LOD
3-Trifluoromethylphenylpiperazine	102.3 ± 8.7	1.8 ± 2.7	4.6	9.3	0.2	0.8	<LOD
1-(3-chlorophenyl) Piperazine	96.7 ± 5.2	−0.4 ± 3.6	4.0	6.7	0.2	0.8	<LOD
Tramadol	91.0 ± 5.1	−10.1 ± 6.6	3.8	6.3	0.2	0.8	<LOD
Fentanyl	100.4 ± 5.7	−1.8 ± 1.9	1.6	3.0	0.2	0.8	<LOD

[a] LOD—limit of detection; [b] LOQ—limit of quantification; [c] RSD—relative standard deviation.

Table S4. The human excretion factors of the target drugs, molecular weight ratio of parent and metabolite and typical dose.

Drug	Selected Biomarker	Excretion Factor (%)	MW_p/MW_m [a]	Typical Dose (mg)
Methamphetamine	Methamphetamine	43 [1]	1.00	30 [2]
Ketamine	Ketamine	16 [b] [2,3]	1.00	75 [2]
MDMA [c]	MDMA	26 [4]	1.00	100 [1]
Codeine	Codeine	30 [5]	1.00	38 [3]
Tramadol	Tramadol	30 [2]	1.00	50 [2]
Cocaine	Benzoylecgonine	29 [6]	1.05	100 [1]
Methadone	EDDP	55 [5]	1.12	25 [4]
Morphine	Morphine	77.7 [7]	1.00	20 [d] [8]
Heroin	Morphine	42 [1]	1.29	15 [9]

[a] MW_p/MW_m—molecular weight ratio of parent and metabolite; [b] Mean excretion factor; [c] MDMA—3,4-Methylenedioxymethamphetamine; [d] Assume the typical dose is same to heroin based on the similar structures;.

Table S5. Number of drug dependents of amphetamine-type stimulants and opiates in Malaysia from 2013–2017 [10].

Year	Opiates	Methamphetamine	Amphetamine-type stimulants tablets [a]
2013	16041	3008	476
2014	14502	5356	1774
2015	16616	8807	1309
2016	16985	12738	3395
2017	10154	14785	5130

[a] Includes methamphetamine, ecstasy type (MDMA) and amphetamine.

References:

1. Zuccato, E.; Chiabrando, C.; Castiglioni, S.; Bagnati, R; Fanelli, R. Estimating community drug abuse by wastewater analysis. *Environ. Health Perspect*. **2008**, *116*, 1027–1032.
2. Yargeau, V.; Taylor, B.; Li, H.; Rodayan, A; Metcalfe, C.D. Analysis of drugs of abuse in wastewater from two Canadian cities. *Sci. Total Environ*. **2014**, *487*, 722–730.
3. Baselt, R. *Disposition of Toxic Drugs and Chemicals in Man*. Biomedical Publications: Foster City, CA, 2008.
4. Postigo, C.; de Alda, M.L.; Barcelo, D. Evaluation of drugs of abuse use and trends in a prison through

wastewater analysis. *Environ. Int*. **2011**, *37*, 49–55.

5. Thai, P.K.; Lai, F.Y.; Bruno, R.; van Dyken, E.; Hall, W.; O'Brien, J.; Prichard, J.; Mueller, J.F. Refining the excretion factors of methadone and codeine for wastewater analysis - Combining data from pharmacokinetic and wastewater studies. *Environ. Int*. **2016**, *94*, 307–314.
6. Castiglioni, S.; Bijlsma, L.; Covaci, A.; Emke, E.; Hernández, F.; Reid, M.; Ort, C.; Thomas, K.V.; van Nuijs, A.L.; de Voogt, P.; et al. Evaluation of Uncertainties Associated with the Determination of Community Drug Use through the Measurement of Sewage Drug Biomarkers. *Environ. Sci. Technol*. **2013**, *47*, 1452–1460.
7. Khan, U.; Nicell, J.A. Refined sewer epidemiology mass balances and their application to heroin, cocaine and ecstasy. *Environ. Int*. **2011**, *37*, 1236–1252.
8. Baker, D.R.; Barron, L.; Kasprzyk-Hordern, B. Illicit and pharmaceutical drug consumption estimated via wastewater analysis. Part A: Chemical analysis and drug use estimates. *Sci. Total Environ*. **2014**, *487*, 629–641.
9. Sulaiman, M.; Kunalan, V.; Yap, A.T.W.; Lim, W.J.L.; Ng, J.J.Y.; Loh, S.W.X.; Chan, K.B. Heroin in Malaysia and Singapore. *Drug Test. Anal*. **2018**, *10*, 109–119.
10. Kanato, M.; Choomwattana, C.; Sarasiri, R.; Leyatikul, P., (Eds.). ASEAN Drug Monitoring Report 2017. Narcotics Cooperation Center ASEAN: Bangkok, Thailand, 2018. Available online: https://asean.org/storage/2016/10/Doc-3-ADM-Report-2017-as-of-16-Aug18-FINAL.pdf (accessed on 17 September 2019).
